# Bolus administration T1 mapping as a marker of interstitial myocardial fibrosis in severe aortic stenosis

**DOI:** 10.1186/1532-429X-14-S1-T4

**Published:** 2012-02-01

**Authors:** Rick Wage, Andrew Jabbour, Tevfik F Ismail

**Affiliations:** 1 CMR Unit, Royal Brompton Hospital, London, UK

## Summary

Post-gadolinium bolus T1 mapping was used to assess interstitial myocardial fibrosis in a group of patients with severe aortic stenosis. The changes identified were correlated with regional contractile perfomance as assessed by CSPAMM tagging. T1 mapping proved to be a practical feasible approach for assessing diffuse fibrosis.

## Background

Aortic stenosis (AS) is associated with increased interstitial myocardial fibrosis (IMF). This is detectable by cardiovascular magnetic resonance (CMR) after gadolinium infusion. T1-mapping-derived partition coefficients (λ) and extracellular volume fractions (Ve) have also been shown to correlate with IMF after a simple bolus administration of gadolinium. We hypothesized that the λ and Ve would also detect interstitial expansion in severe AS patients compared to healthy controls and that these measures would correlate with abnormal myocardial strain using a high temporal-resolution tagging sequence.

## Methods

A Modified Look-Locker Inversion Recovery (MOLLI) sequence was used to generate eleven T1-weighted images. Myocardial and blood pool T1 values were derived by fitting a signal intensity-time curve using CMR42®. The λ was determined by plotting (1/T1myo vs. 1/T1blood pool) at various time points once contrast equilibrium was reached. Ve was derived accounting for the hematocrit. Ventricular long-axis and short-axis T1 maps (basal, mid-ventricular and apical) were acquired using a 1.5T scanner (Siemens) before and 1,2,5,8,15,20,25 and 30 minutes after contrast. Myocardial tagging images were acquired using both single- and multiple-breath-hold Complementary Spatial Modulation of Magnetization (CSPAMM) sequences in multiple planes and analysed with inTag® (Lyon, France).

## Results

Subjects with severe AS displayed higher λ and Ve (p=0.02). The λ and Ve correlated with indices of reduced myocardial function including reduced strain (p<0.05) and increased left atrial dilatation (p=0.001). In this presentation, the tips and pitfalls of T1 mapping using MOLLI will be discussed, including detailed discussion of imaging planes, arrhythmia management, breath-hold times, gadolinium administration and artefact reduction.

## Conclusions

T1-mapping-derived λ and Ve are significantly elevated in patients with AS compared to healthy controls and correlate well with indices of reduced myocardial performance. This difference was quantifiable after a simple bolus administration of gadolinium. T1-mapping λ and Ve derivation after bolus gadolinium administration is clinically practical and holds promise for the detection of IMF in severe AS.

## Funding

This project was supported by the NIHR Royal Brompton Cardiovascular Biomedical Research Unit.

